# Factors associated with peripheral neuropathy development during gemcitabine plus albumin-bound paclitaxel therapy as first-line treatment for unresectable pancreatic cancer: a retrospective evaluation

**DOI:** 10.1186/s40780-026-00572-4

**Published:** 2026-04-07

**Authors:** Tomoya Ban, Makoto Miura

**Affiliations:** Department of Pharmacy, Rakuwakai Otowa Hospital, Yamashina-ku, Kyoto, 607-8062 Japan

**Keywords:** Chemotherapy-induced peripheral neuropathy, Gemcitabine, Nab-paclitaxel, Pancreatic cancer, Risk factors

## Abstract

**Background:**

Gemcitabine plus albumin-bound paclitaxel is widely used as first-line therapy for unresectable pancreatic cancer, but it is frequently complicated by chemotherapy-induced peripheral neuropathy, which may lead to functional impairment and treatment modification. Factors associated with clinically relevant neuropathy, defined as grade 2 or higher, remain insufficiently clarified. This study aimed to identify factors associated with the development of grade ≥ 2 neuropathy during therapy.

**Methods:**

We conducted a retrospective observational study using medical records of patients with unresectable pancreatic cancer who received this combination therapy at Rakuwakai Otowa Hospital from April 2015 to March 2024. Patient characteristics, treatment details, and neuropathy outcomes were collected. Univariate and multivariate logistic regression analyses were performed to evaluate factors associated with grade ≥ 2 neuropathy. Statistical significance was defined as *P* < 0.05.

**Results:**

Fifty-seven patients were included in the analysis, and 19 patients (33.3%) developed grade ≥ 2 neuropathy. In multivariate logistic regression, a history of diabetes mellitus was significantly associated with the development of grade ≥ 2 neuropathy (odds ratio 3.89, 95% confidence interval 1.06–14.3, *P* = 0.041). No other clinical or treatment-related variables demonstrated significant associations.

**Conclusions:**

A history of diabetes mellitus may be associated with the development of grade ≥ 2 CIPN in patients with unresectable pancreatic cancer undergoing GnP therapy. Healthcare professionals should detect CIPN symptoms early and manage them appropriately, particularly in patients with diabetes mellitus.

**Supplementary Information:**

The online version contains supplementary material available at 10.1186/s40780-026-00572-4.

## Background

In 2022, pancreatic cancer was the sixth leading cause of cancer-related death worldwide [1] and the fourth leading cause in Japan [2]. Pancreatic cancer has a poor prognosis; its 5-year survival rate is 8.5% in Japan [2], and approximately 80% of cases are diagnosed at an unresectable stage [3].

First-line treatment for unresectable pancreatic cancer is chemotherapy [4]. Previously, gemcitabine (GEM) monotherapy was the standard chemotherapy regimen [5]; however, GEM plus albumin-bound paclitaxel (nanoparticle albumin-bound paclitaxel [nab-PTX]) (GnP) therapy has become one the main first-line options because it provides longer overall survival [6].

Reported adverse events of GnP therapy include myelosuppression, nausea, fatigue, alopecia, and chemotherapy-induced peripheral neuropathy (CIPN) [6]. CIPN is a frequent adverse event that affects the quality of life and often leads to dose reduction or treatment discontinuation. In clinical trials of GnP therapy for pancreatic cancer, grade 3 CIPN has been defined as the criterion for dose modification [6]; however, in clinical trials of carboplatin plus nab-PTX therapy for lung cancer, grade 2 CIPN has been used as the criterion [7]. Additionally, in real-world practice, dose reduction is often initiated when grade ≥ 2 CIPN develops, even in patients with pancreatic cancer, after considering the general condition of the patient [8].

Advanced age and more frequent chemotherapy cycles have been associated with CIPN development during GnP therapy [9]. However, most previous studies evaluated predictors of grade ≥ 1 CIPN. More recently, studies focusing on grade ≥ 2 CIPN have identified baseline peripheral neuropathy as an independent factor associated with its development [10]. However, these studies included patient populations containing second-line cases. To date, no study has specifically evaluated risk factors for grade ≥ 2 CIPN exclusively in patients receiving first-line GnP therapy.

Therefore, we retrospectively investigated factors associated with grade ≥ 2 CIPN development in patients undergoing first-line GnP therapy for unresectable pancreatic cancer.

## Methods

### Study population

Patients with unresectable pancreatic cancer who received GnP therapy as first-line treatment at Rakuwakai Otowa Hospital between April 1, 2015, and April 30, 2024, were included in this study. Patients who discontinued treatment within one cycle and those younger than 18 years were excluded. The standard GnP regimen consisted of nab-PTX 125 mg/m² and GEM 1,000 mg/m² administered on days 1, 8, and 15 of a 28-day cycle, with day 22 as a rest day [6].

### Outcomes and data collection

Patient background characteristics were collected from electronic medical records. These included age at chemotherapy initiation, sex, body mass index, history of diabetes mellitus, smoking history, alcohol consumption, performance status, concomitant medications, history of breast cancer, endocrine therapy, and presence of distant metastasis. Diabetes mellitus was defined as documentation of diabetes mellitus by a physician in the medical records. Diabetes mellitus cases comprised both a history of diabetes mellitus and ongoing diabetes mellitus as a comorbidity. Concomitant medications included CYP2C8 inhibitors, CYP3A4 inhibitors, drugs commonly used for the management of peripheral neuropathy, and drugs known to cause neuropathy. Concomitant medications with the potential to induce peripheral neuropathy were evaluated to account for possible confounding factors associated with its development; however, they were not evaluated to assess their impact on the pharmacokinetics of nab-PTX. Laboratory data obtained before the initiation of chemotherapy included the white blood cell count, platelet count, hemoglobin level, aspartate aminotransferase level, alanine aminotransferase level, total bilirubin level, serum albumin level, C-reactive protein level, blood urea nitrogen level, serum creatinine level, and estimated glomerular filtration rate.

### Evaluation of CIPN severity

CIPN severity was assessed based on the National Cancer Institute Common Terminology Criteria for Adverse Events (version 5.0) for peripheral neuropathy (peripheral motor neuropathy and peripheral sensory neuropathy) [11] using the grading documented in the medical records. At Rakuwakai Otowa Hospital, routine grading of adverse events includes the completion of a self-assessment of adverse events by the patient at each visit. Thereafter, a pharmacist or nurse interviews the patient based on the results of the self-assessment to confirm the validity of the reported grades. When discrepancies between the self-assessment results and the results of the evaluation by the healthcare provider are identified, grading is corrected as necessary.

### Definitions

Drugs with package insert descriptions that indicated CYP2C8 inhibitory effects and those with reported CYP2C8 inhibitory effects were considered CYP2C8 inhibitors [12, 13]. CYP3A4 inhibitors were defined based on their inhibition values reported by Ohno et al. [14]. Specific agents included in this category are listed in Supplemental Table S1. Drugs with potential for the treatment of CIPN were defined according to the recommendations of the European Society for Medical Oncology guidelines for neuropathy management [15]. Drugs with the potential to cause peripheral neuropathy were defined according to previous reports [16]. Specific drugs included in each concomitant medication category are provided in Supplemental Table S1.

### Statistical analysis

Patients were classified into two groups: those who developed grade ≥ 2 CIPN and those who did not. For patients who developed grade ≥ 2 CIPN, the number of treatment cycles and cumulative dose of nab-PTX at the time of onset were evaluated. Grade ≥ 2 CIPN was used as the dependent variable, and patient background characteristics (age, sex, body mass index, Eastern Cooperative Oncology Group performance status, history of diabetes mellitus, smoking history, alcohol consumption, history of breast cancer, endocrine therapy, and presence of distant metastasis), laboratory data (white blood cell count, platelet count, hemoglobin level, aspartate aminotransferase level, alanine aminotransferase level, total bilirubin level, serum albumin level, C-reactive protein level, blood urea nitrogen level, serum creatinine level, estimated glomerular filtration rate), and concomitant medication use (CYP2C8 inhibitors, CYP3A4 inhibitors, drugs potentially used for CIPN management, and drugs potentially causing peripheral neuropathy) were included as independent variables in the univariate analysis. The Mann–Whitney U test was used for continuous variables, and Fisher’s exact test was used for categorical variables.

In the multivariate logistic regression analysis, age [9], history of diabetes mellitus [17], and sex [18], which are associated with CIPN induced by taxane-based chemotherapy, were included as independent variables. Risk factors for taxane-induced CIPN include baseline pre-existing neuropathy, age, sex, diabetes mellitus, and treatment cycle number [9, 10, 17, 18]. Because the present study was restricted to patients receiving first-line GnP therapy, pre-existing neuropathy related to prior chemotherapy was not applicable. Additionally, treatment cycle number is a time-dependent factor that is determined after treatment initiation; therefore, it was not included because the primary objective of this study was pretreatment risk stratification. Accordingly, age, sex, and history of diabetes mellitus were selected a priori because these factors are assessable before treatment initiation and supported by previous evidence and biological plausibility. A common recommendation that is suggested to ensure model stability is the inclusion of 10 or more outcome events per variable in multivariable logistic regression. However, methodological studies have suggested that this threshold is not absolute, and that models with fewer than 10 events per variable may provide reasonably stable estimates under certain conditions [19]. Therefore, to reduce the risk of overfitting because of the limited number of outcome events, the explanatory variables were restricted to three prespecified variables (age, sex, and history of diabetes mellitus).

Kaplan–Meier curves were constructed as exploratory post hoc analyses to evaluate time to the first occurrence of grade ≥ 2 CIPN, and patients with and without diabetes mellitus were compared using the log-rank test. Patients without grade ≥ 2 CIPN development were censored at the date of the last administration of GnP. A multivariable Cox proportional hazards model including age, sex, and history of diabetes mellitus was additionally performed to evaluate time to the first occurrence of grade ≥ 2 CIPN. These covariates were selected a priori based on previous evidence and were identical to those used in the multivariable logistic regression analysis. The proportional hazards assumption was assessed using Schoenfeld residuals.

Clinical laboratory variables with missing data were excluded from the univariate analysis. Statistical analyses were performed using EZR (version 1.67) [20].

## Results

### Patient characteristics

A total of 68 patients with unresectable pancreatic cancer received GnP therapy as first-line treatment at Rakuwakai Otowa Hospital. Of these, 11 patients who discontinued treatment within one cycle were excluded, leaving 57 patients for the final analysis. No patients under the age of 18 were included. Among the eligible patients, 29 (50.9%) were female, and the median age was 70 years. Fourteen patients (24.6%) had comorbid diabetes mellitus. Seven patients (12.3%) received concomitant CYP2C8 inhibitors, which are involved in the metabolism of nab-PTX, while no patients received CYP3A4 inhibitors. Seventeen patients (29.8%) received concomitant medications potentially used for the treatment of CIPN (Table 1).

**Table 1:** Patient characteristics

Characteristics	Total (*n* = 57)	
Age (years)^b^	70	(52–82)
Female^a^	29	(50.9%)
BMI (kg/m²)^b^	21.9	(15.5–33.5)
Performance status (0/1/2)^a^	19/38/0	
Diabetes mellitus^a^	14	(24.6%)
Smoking history^a^	25	(43.9%)
Alcohol consumption^a^	30	(52.6%)
Concomitant use of CYP2C8 inhibitors^a^	7	(12.3%)
Concomitant use of CYP3A4 inhibitors^a^	0	(0%)
Concomitant use of drugs potentially used for CIPN treatment^a^	17	(29.8%)
Concomitant use of drugs potentially causing peripheral neuropathy^a^	0	(0%)

a Data are expressed as n (%)

Data are expressed as median (range)

Abbreviations: BMI, body mass index; PS, performance status; CIPN, chemotherapy-induced peripheral neuropathy

**Table 2:** Incidence and severity of CIPN

Variables	Total (*n* = 57)
Grade 0	17 (29.8%)
All-Grade	40 (70.2%)
└─ Grade 1^a^	└─ 21 (36.8%)
└─ Grade ≥ 2	└─ 19 (33.3%)
└─ Grade 2^a^	└─ 16 (28.1%)
└─ Grade 3^a^	└─ 3 (5.3%)
Number of treatment cycles until onset of grade ≥ 2 CIPN^b^	5 (3–13)
Cumulative nab-PTX dose until onset of grade ≥ 2 CIPN (mg/m²)^b^	1,800 (680–4,200)

a Data are expressed as n (%)

b Data are expressed as median (range)

Abbreviations: CIPN, chemotherapy-induced peripheral neuropathy; nab-PTX, nanoparticle albumin-bound paclitaxel

### Incidence and severity of CIPN

Among the 57 patients who received GnP therapy, 40 (70.2%) developed CIPN. The CIPN severity distribution was as follows: grade 1, 21 (36.8%) patients; grade 2, 16 (28.1%) patients; and grade 3, 3 (5.3%) patients. Grade ≥ 2 CIPN developed in 19 (33.3%) patients. At the time of maximal CIPN severity, these 19 patients had received a median of 5 treatment cycles and median cumulative nab-PTX dose of 1,800 mg/m^2^ (Table 2).

### Factors associated with grade ≥ 2 CIPN

To identify factors associated with grade ≥ 2 CIPN development during GnP therapy, univariate analyses were performed to examine the relationship between baseline patient characteristics or laboratory parameters at the initiation of chemotherapy and the occurrence of grade ≥ 2 CIPN. Serum albumin values of six patients were missing; therefore, they were not included in the univariate analysis. Additionally, the medical records did not indicate that any patients were receiving concomitant CYP3A4 inhibitors or drugs with the potential to cause peripheral neuropathy; therefore, these variables were not included in the univariate analysis. These analyses revealed that the presence of diabetes mellitus was statistically significantly associated with grade ≥ 2 CIPN (*P* = 0.049) (Table 3).

**Table 3:** Univariate analysis of factors associated with grade ≥ 2 CIPN

Variables	grade ≥ 2 CIPN (*n* = 19)	No grade ≥ 2 CIPN (*n* = 38)	*p*-Value
Age (years)^b^	71 (53–82)	69.5 (52–81)	1.00
Female^a^	8 (42.1%)	21 (55.3%)	0.41
BMI (kg/m²) ^b^	22.5 (16.9–31.6)	20.8 (15.5–33.5)	0.052
Performance status (1/0)^a^	11/8	27/11	0.38
Diabetes mellitus^a^	8 (42.1%)	6 (15.8%)	0.049
Smoking history^a^	12 (63.1%)	13 (34.2%)	0.050
Alcohol consumption^a^	10 (52.6%)	20 (52.6%)	1.00
WBC (≥ Grade 1)^a^	0 (0%)	6 (7.9%)	0.16
Plt (≥ Grade 1)^a^	2 (10.5%)	1 (2.6%)	0.26
Hb (≥ Grade 1)^a^	3 (15.8%)	7 (18.4%)	1.00
AST (≥ Grade 1)^a^	3 (15.8%)	11 (28.9%)	0.34
ALT (≥ Grade 1)^a^	6 (31.6%)	10 (26.3%)	0.76
T-Bil (≥ Grade 1)^a^	6 (31.6%)	8 (21.1%)	0.52
CRP (mg/dL)^b^	0.78 (0.02–4.49)	0.16 (0.02–9.46)	0.058
BUN (mg/dL)^b^	12.70 (5.2–23.6)	12.25 (6.1–31)	0.97
SCr (mg/dL)^b^	0.64 (0.5–1.06)	0.67 (0.41–0.94)	0.55
eGFR (mL/min/1.73 m²)^b^	76 (41–122)	78.50 (46–114)	0.52
Concomitant use of CYP2C8 inhibitors^a^	1 (5.3%)	6 (15.8%)	0.41
Concomitant use of drugs potentially used for CIPN treatment^a^	5 (26.3%)	12 (31.6%)	0.77
History of breast cancer	1 (5.3%)	2 (5.3%)	1.00
Endocrine therapy	1 (5.3%)	2 (5.3%)	1.00
Distant metastasis	8 (42.1%)	12 (31.6%)	0.56

a Data are expressed as n (%)

b Data are expressed as median (range)

Continuous variables were analyzed using the Mann–Whitney U test, and categorical variables were analyzed using Fisher’s exact test

A multivariate logistic regression analysis including age, sex, and diabetes mellitus as explanatory variables revealed that diabetes mellitus was an independent and significant factor associated with grade ≥ 2 CIPN (odds ratio, 3.89; 95% confidence interval, 1.06–14.3; *P* = 0.041). No other variables were significantly associated with grade ≥ 2 CIPN (Table 4).

**Table 4:** Multivariate logistic regression analysis of factors associated with grade ≥ 2 CIPN

Variables	Odds ratio	95%CI	*p*-Value
Female (Ref. male)	0.68	0.21–2.21	0.53
Age (years)	0.97	0.90–1.06	0.49
Diabetes mellitus (Ref. none)	3.89	1.06–14.3	0.041

Multivariate logistic regression analysis was performed to identify independent factors associated with grade ≥ 2 CIPN

In the exploratory post hoc time-to-event analysis, Kaplan–Meier curves showed that grade ≥ 2 CIPN developed significantly earlier in patients with diabetes mellitus than in those without diabetes mellitus (log-rank *p* = 0.004) (Supplemental Figure S1). In the multivariable Cox proportional hazards analysis including age, sex, and history of diabetes mellitus, history of diabetes mellitus was independently associated with an increased hazard of grade ≥ 2 CIPN (hazard ratio, 3.98; 95% confidence interval, 1.45–10.93; *p* = 0.007) (Supplemental Table S2).

## Discussion

In this retrospective study of unresectable pancreatic cancer treated with first-line GnP therapy, a history of diabetes mellitus was identified as an independent factor associated with grade ≥ 2 CIPN. In clinical trials evaluating GnP therapy, the incidence of CIPN has been reported as a safety outcome. In international phase I/II and phase III trials, the incidence of CIPN of any grade was 85.3% and 54.0%, respectively, and the incidence of grade ≥ 2 CIPN was 31.1% in an international phase III trial [6, 21, 22]. In this study, the incidence rates of all grades of CIPN and grade ≥ 2 CIPN were 70.2% and 33.3%, respectively, indicating generally consistent results.

Previous studies included mixed populations of patients receiving first-line and second-line treatment [10]. Because prior treatment may influence the presence or severity of peripheral neuropathy, findings from such populations may not be directly generalizable to patients receiving first-line therapy. In contrast, the present study exclusively focused on patients receiving first-line GnP therapy and evaluated factors associated with grade ≥ 2 CIPN development, identifying a history of diabetes mellitus as an independent associated factor. These findings clarify the risk factors for grade ≥ 2 CIPN specifically within a first-line treatment setting.

The significant association between a history of diabetes mellitus and grade ≥ 2 CIPN in this study is consistent with the findings of previous studies of taxane-based chemotherapy [17]. Nab-PTX is a microtubule inhibitor that is considered to induce CIPN through impairment of axonal transport [23]; however, diabetic neuropathy is also primarily characterized by axonal damage [24, 25], suggesting that shared pathophysiological mechanisms may contribute to grade ≥ 2 CIPN development. Baseline HbA1c levels in patients with diabetes mellitus did not show marked deterioration during treatment (data not shown). A review of the medical records revealed no documented symptoms suggestive of peripheral neuropathy, such as numbness, or clinician-recorded neuropathy at the time of GnP therapy initiation among patients with a history of diabetes mellitus.

Assessments of CIPN severity are often subject to discrepancies between healthcare providers and patients [26], and mild symptoms may be difficult to detect using the Common Terminology Criteria for Adverse Events. Patient-reported outcomes can enable early detection of such symptoms and functional impairments [27]. Therefore, incorporating systematic patient-reported outcomes-based assessments of patients with diabetes mellitus may be particularly valuable to detecting CIPN and providing timely intervention.

Previous studies reported that body mass index [28], smoking history [29], female sex [18], and older age [9] are associated with CIPN development. There variables were also examined during the present study; however, sex and age were not significantly associated with grade ≥ 2 CIPN. Although body mass index and smoking history were not statistically significant, both showed trends that suggested an association consistent with previous findings. Similarly, the C-reactive protein level also showed a trend toward an association, but a definitive conclusion could not be drawn. These discrepancies may be attributable to the restriction of our analysis to grade ≥ 2 CIPN and differences in assessment methods and patient populations.

Because the concomitant use of CYP2C8 inhibitors with paclitaxel can increase the risk of CIPN [30], we also analyzed the effect of the concomitant use of CYP2C8 or CYP3A4 inhibitors, which may influence the metabolism of nab-PTX. However, a clear association with grade ≥ 2 CIPN was not observed in patients who received CYP2C8 inhibitors, and no patients received CYP3A4 inhibitors. Additionally, the concomitant use of drugs for the treatment of CIPN and the use of drugs known to cause peripheral neuropathy were investigated, but no associations with grade ≥ 2 CIPN were identified. These findings suggest that the clinical impact of concomitant medications on the development of CIPN is limited.

Cumulative dose is a well-established risk factor for taxane-induced CIPN. Because cumulative dose increases with continued treatment, it was not included as an explanatory variable in the primary multivariable model, which focused on pretreatment risk stratification. In exploratory analyses, the cumulative nab-PTX dose at the time of grade ≥ 2 CIPN onset did not differ significantly according to history of diabetes mellitus (median 1,700 mg/m² in patients without diabetes mellitus vs. 2,130 mg/m² in those with diabetes mellitus; Mann–Whitney U test, *p* = 0.409). In addition, exploratory time-to-event analyses demonstrated that patients with diabetes mellitus developed grade ≥ 2 CIPN earlier than those without diabetes mellitus. A multivariable Cox proportional hazards model also demonstrated an increased hazard of grade ≥ 2 CIPN associated with history of diabetes mellitus. Taken together, these findings suggest that the association between diabetes mellitus and grade ≥ 2 CIPN cannot be explained solely by differences in cumulative exposure. However, given the limited sample size and retrospective design, these findings should be interpreted cautiously. Further prospective studies incorporating detailed cumulative exposure modeling are warranted.

This study had some limitations. First, because this was a single-center, retrospective, observational study, the sample size and number of grade ≥ 2 CIPN events were limited; therefore, the stability of the multivariate estimates and generalizability of the findings may have been affected. Second, because of the retrospective design, residual and unmeasured confounding factors related to incomplete clinical information could not be fully excluded. Although documented symptoms suggestive of peripheral neuropathy were not identified at the time of GnP therapy initiation in patients with diabetes mellitus, subtle or subclinical neuropathy that was not documented in the medical records may have been under-recognized in routine clinical practice. Additionally, the severity of diabetes mellitus, glycemic control status, and comorbidities that may influence peripheral neuropathy were not sufficiently evaluated and may have affected the interpretation of the results. In addition, serum vitamin D levels were not routinely measured, and therefore vitamin D insufficiency, which has been reported as a potential risk factor for taxane-induced CIPN [31], could not be evaluated. Third, CIPN was assessed based on National Cancer Institute Common Terminology Criteria for Adverse Events information recorded in the medical records without re-evaluation by a physician, and potential variability in assessment procedures or inter-rater differences may have influenced the results. Therefore, the present findings should be interpreted with appropriate caution. Future studies that incorporate prospective designs and more detailed cumulative exposure assessments may further strengthen and extend these findings. Despite these limitations, this study demonstrated that a history of diabetes mellitus was associated with grade ≥ 2 CIPN development during first-line GnP therapy, thus providing clinically important insights.

## Conclusions

A history of diabetes mellitus is associated with grade ≥ 2 CIPN development in patients with unresectable pancreatic cancer receiving first-line GnP therapy. Early detection and appropriate management of CIPN symptoms are essential for such patients. Future studies should elucidate the pathophysiological mechanisms by which diabetes mellitus contributes to CIPN progression and establish preventive and therapeutic strategies accordingly.

## Supplementary Information

Below is the link to the electronic supplementary material.

Supplementary Material 1

Supplementary Material 2: Supplemental Figure S1. Kaplan–Meier curves for time to grade ≥ 2 CIPN stratified by the history of diabetes mellitus. Abbreviations: CIPN, chemotherapy-induced peripheral neuropathy; GnP, gemcitabine plus nanoparticle albumin-bound paclitaxel.

## Data Availability

The datasets generated and analyzed during the current study are not publicly available because they contain personal clinical information of the patients. Data may be available from the corresponding author on reasonable request and with the permission of Rakuwakai Otowa Hospital.

## Abbreviations

Alb: Albumin
ALT: Alanine aminotransferase
AST: Aspartate aminotransferase
BMI: Body Mass Index
BUN: Blood Urea Nitrogen
CBDCA: Carboplatin
CIPN: Chemotherapy-Induced Peripheral Neuropathy
CRP: C-Reactive Protein
eGFR: Estimated Glomerular Filtration Rate
ESMO: European Society for Medical Oncology
GEM: Gemcitabine
GnP: Gemcitabine plus nab-Paclitaxel
Hb: Hemoglobin
NSAIDs: Non-Steroidal Anti-Inflammatory Drugs
OS: Overall Survival
Plt: Platelet count
PRO: Patient-Reported Outcome
PS: Performance Status
QOL: Quality of Life
SCr: Serum Creatinine
T-Bil: Total Bilirubin
WBC: White Blood Cells
