# Supplementary material for: Factors associated with peripheral neuropathy development during gemcitabine plus albumin-bound paclitaxel therapy as first-line treatment for unresectable pancreatic cancer: a retrospective evaluation
Source: J Pharm Health Care Sci. 2026 Apr 7;12:52. doi: 10.1186/s40780-026-00572-4 (PMC13188612; doi:10.1186/s40780-026-00572-4)
Supplement: Supplementary file 1 — Supplementary Material 1 [file 40780_2026_572_MOESM1_ESM.docx]

Table S1. List of specific drugs included in each concomitant medication category

| Category | Specific drugs included |
| --- | --- |
| CYP2C8 inhibitors | candesartan cilexetil, clopidogrel, sulfamethoxazole-trimethoprim, selpercatinib, tazemetostat, deferasirox, lascufloxacin |
| CYP3A4 inhibitors | ketoconazole, voriconazole, itraconazole, clarithromycin, erythromycin, diltiazem, fluconazole, verapamil |
| Drugs potentially used for CIPN treatment | goshajinkigan, vitamin B12, NSAIDs, acetaminophen, tramadol, opioids, pregabalin, mirogabalin, duloxetine, gabapentin, amitriptyline, nortriptyline, imipramine |
| Drugs potentially causing peripheral neuropathy | isoniazid, ethambutol, metronidazole, antiretroviral drugs, statins, phenytoin, tacrolimus, colchicine, amiodarone, interferon |

Table S2. Multivariable Cox proportional hazards analysis for grade ≥2 CIPN

| Variables | Odds ratio | 95%CI | p-Value |
| --- | --- | --- | --- |
| Female (Ref. male) | 1.20 | 0.46–3.10 | 0.71 |
| Age (years) | 1.03 | 0.97–1.10 | 0.31 |
| Diabetes mellitus (Ref. none) | 3.98 | 1.45–10.93 | 0.007 |

*Multivariable Cox proportional hazards analysis was performed to evaluate time to the first occurrence of grade ≥2 CIPN.*
