# Supplementary material for: Factors associated with peripheral neuropathy development during gemcitabine plus albumin-bound paclitaxel therapy as first-line treatment for unresectable pancreatic cancer: a retrospective evaluation
Source: J Pharm Health Care Sci. 2026 Apr 7;12:52. doi: 10.1186/s40780-026-00572-4 (PMC13188612; doi:10.1186/s40780-026-00572-4)
Supplement: Supplementary file 2 — Supplementary Material 2: Supplemental Figure S1. Kaplan–Meier curves for time to grade ≥ 2 CIPN stratified by the history of diabetes mellitus. Abbreviations: CIPN, chemotherapy-induced peripheral neuropathy; GnP, gemcitabine plus nanoparticle albumin-bound paclitaxel. [file 40780_2026_572_MOESM2_ESM.pptx]

## Slide 1
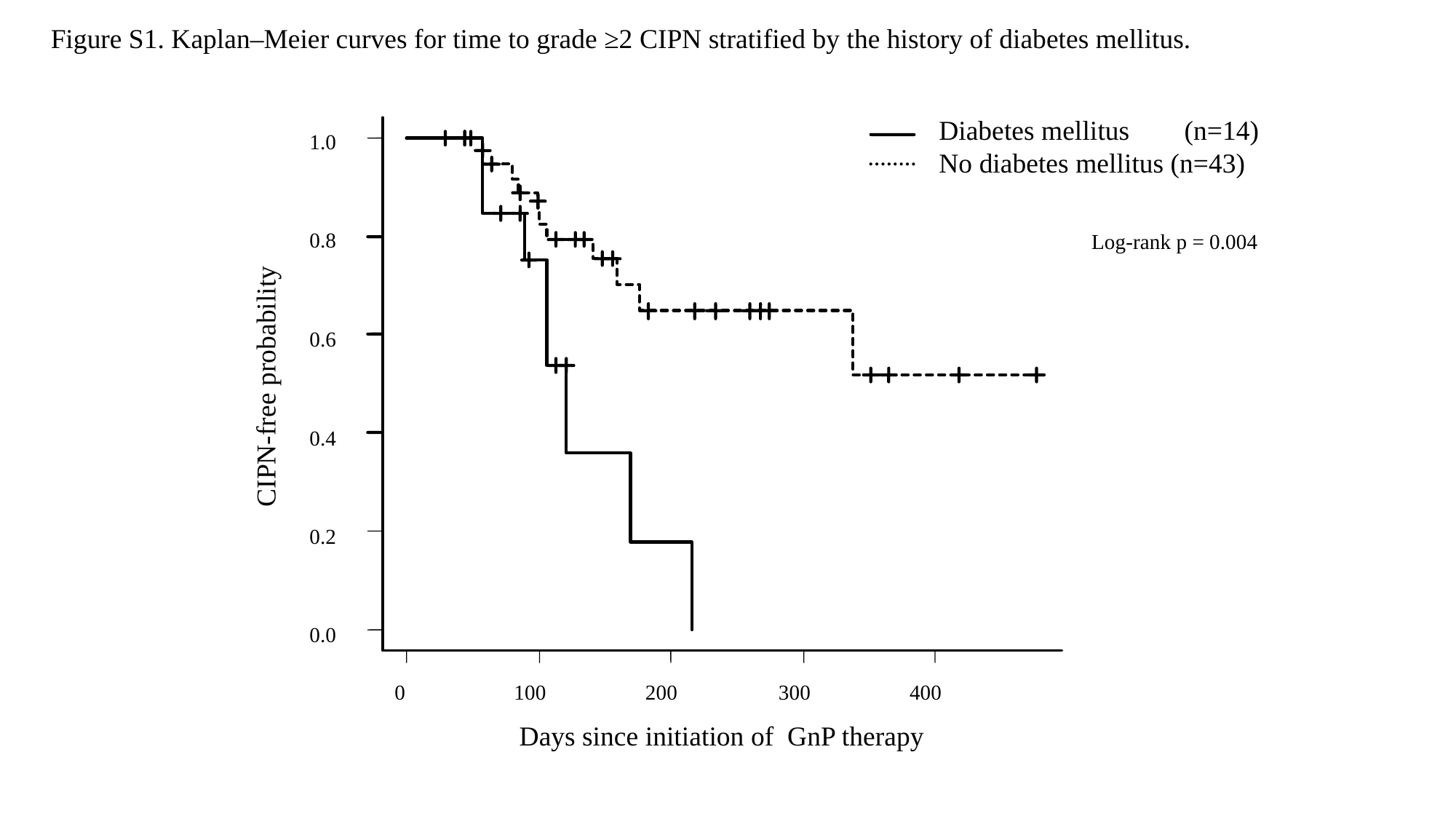

Figure S1. Kaplan–Meier curves for time to grade ≥2 CIPN stratified by the history of diabetes mellitus.
Diabetes mellitus　 (n=14)
1.0
No diabetes mellitus (n=43)
0.8
Log-rank p = 0.004
0.6
CIPN-free probability
0.4
0.2
0.0
0
100
200
300
400
Days since initiation of GnP therapy
